# Metformin decreases the incidence of ovarian hyperstimulation syndrome: an experimental study

**DOI:** 10.1186/1757-2215-6-62

**Published:** 2013-09-08

**Authors:** Evelin M Elia, Ramiro Quintana, Carlos Carrere, María V Bazzano, Gastón Rey-Valzacchi, Dante A Paz, María C Pustovrh

**Affiliations:** 1 Laboratorio de Biología del Desarrollo, Facultad de Ciencias Exactas y Naturales, Instituto de Fisiología, Biología Molecular y Neurociencias (IFIBYNE-CONICET-UBA), Pabellón 2, 4 C1428EHA Cdad Universitaria, Buenos Aires, Argentina; 2 Procrearte, Medicina Reproductiva y Molecular, Buenos Aires, Argentina; 3 Departamento de Biodiversidad y Biología Experimental, Facultad de Ciencias Exactas y Naturales, Universidad de Buenos Aires, Pabellón 2, Cdad, Universitaria, Buenos Aires, Argentina; 4 Departamento de Morfología, Escuela de Ciencias Básicas, Facultad de Salud, Universidad del Valle, Cali, Colombia

## Abstract

**Background:**

In assisted reproduction cycles, gonadotropins are administered to obtain a greater number of oocytes. A majority of patients do not have an adverse response; however, approximately 3-6% develop ovarian hyperstimulation syndrome (OHSS). Metformin reduces the risk of OHSS but little is known about the possible effects and mechanisms of action involved.

**Objective:**

To evaluate whether metformin attenuates some of the ovarian adverse effects caused by OHSS and to study the mechanisms involved.

**Material and methods:**

A rat OHSS model was used to investigate the effects of metformin administration. Ovarian histology and follicle counting were performed in ovarian sections stained with Masson trichrome. Vascular permeability was measured by the release of intravenously injected Evans Blue dye (EB). VEGF levels were measured by commercially immunosorbent assay kit. COX-2 protein expression was evaluated by western blot and NOS levels were analyses by immunohistochemistry.

**Results:**

Animals of the OHSS group showed similar physiopathology characteristics to the human syndrome: increased body weight, elevated progesterone and estradiol levels (P<0.001), increased number of corpora lutea (P<0.001), higher ovarian VEGF levels and vascular permeability (P<0.001 and P<0.01); and treatment with metformin prevented this effect (OHSS+M group; P<0.05). The vasoactive factors: COX-2 and NOS were increased in the ovaries of the OHSS group (P<0.05 and P<0.01) and metformin normalized their expression (P<0.05); suggesting that metformin has a role preventing the increased in vascular permeability caused by the syndrome.

**Conclusion:**

Metformin has a beneficial effect preventing OHSS by reducing the increase in: body weight, circulating progesterone and estradiol and vascular permeability. These effects of metformin are mediated by inhibiting the increased of the vasoactive molecules: VEGF, COX-2 and partially NOS. Molecules that are increased in OHSS and are responsible for a variety of the symptoms related to OHSS.

## Introduction

In assisted reproduction cycles, gonadotropins are administered to obtain a greater number of oocytes. A majority of patients do not have an adverse response; however, approximately 3-6% develop ovarian hyperstimulation syndrome (OHSS), which is characterized by a variety of manifestations including ascites, pleural hemorrhage, hemoconcentration and oliguria. In the severe OHSS, a form of thromboembolism can occur, and without medical care, it may lead to death [1,2].

The main characteristic of these manifestations is an increase in vascular permeability caused by the release of hCG mediators; however, this mechanism is not fully understood. Among the wide variety of angiogenic and vasoactive molecules, vascular endothelial growth factor (VEGF) plays a preponderant role. The production of VEGF in endothelial cells is increased by treatment with hCG, and in turn, the growth factor increases vascular permeability [1]. In the ovary, VEGF is produced in teca and granulose cells. There have been reports showing a relationship between serum VEGF levels and the administration of hCG, suggesting that it can be used as an OHSS predictor [2-4]. Moreover, Gomez et al. has suggested that targeting VEGF and its receptor was an effective preventive approach to treat the syndrome [5,6]. In the mouse skin, Fujii et al. have described that the vascular permeability induced by VEGF is mediated by the local production of nitric oxide (NO) and arachidonic acid metabolites, which are mainly produced by NO synthase (NOS) and cyclooxygenase type 2 (COX-2), respectively [7].

We have previously demonstrated that a beneficial effect of meloxicam (a COX-2 inhibitor) in OHSS is the reduction of ovarian weight and VEGF expression in a rat model of OHSS (Quintana et al., 2008). However, the administration of meloxicam during *in vitro* fertilization procedures is not recommended because it blocks ovulation by inhibiting the breakdown of mature ovarian follicles [8,9].

Metformin (*N,N*-dimethylbiguanide) is one of the most common drugs used for the treatment of type 2 diabetes. Among the various actions of metformin are its effect activating the adenosine 3’5’-monophosphate (AMP) dependent kinase alpha (AMPK-a) pathway, decreasing the glucose production, increasing fatty acid oxidation and promoting the uptake of glucose in cells [10-13]. In polycystic ovary syndrome (PCOS), metformin decreases androgen levels, improves the frequency of ovulation and menstrual cycles, normalizes uterine homeostasis and prevents miscarriages [14-17]. Metformin administration also increases the embryo quality, the clinical pregnancy rate as well as the implantation rate [18], and it is important to highlight that the mechanism of action of metformin involves the modulation of NOS and COX-2 expression in the ovary and the uterus [19-21].

In contrast, it has been demonstrated that PCOS patients undergoing fertility treatment who receive gonadotrophins have an increased risk for OHSS. Whereas, when those women were treated with metformin, the risk of OHSS was reduced [22]. However, little is known about the possible effects and mechanisms by which metformin reduces the risk of OHSS.

In view of these findings, metformin appears to be an alternative treatment for the prevention of OHSS; however, there are no reports that take in consideration the mechanisms by which metformin regulates ovarian physiology in OHSS. Therefore, the purpose of this study is to evaluate whether metformin attenuates some of the ovarian adverse effects in OHSS in an animal model and to study the mechanisms involved.

## Materials and methods

### Study design

Immature female Wistar rats were obtained from Bioterio Central, Facultad de Ciencias Exactas y Naturales, UBA. All research animals were treated in compliance with the guidelines for the care and use of animals approved by our institutions in accordance with the principles of laboratory animal care (NIH Guide for the Care and Use of Laboratory Animals, Institute of Laboratory Animal Resources, National Research Council, Washington, D.C.). Rats were fed with a standard diet, allowed free access to water, and had a 12-hour light cycle (lights-on 7AM to 7PM).

Thirty female rats (weight: 44–50 g) were divided into three groups: (1) the control group (n=10), which received 10 IU of pregnant mare serum gonadotropin (PMSG; Sigma Chemical Co) on the 39th day and 10 IU of hCG (Sigma) 48 hours later (day 41) to mimic routine ovarian stimulation; (2) the OSSH group (n=10), which received 50 IU of PMSG daily from days 37 to 40 and 30 IU hCG on day 41 to induce OHSS; (3) the OHSS+MET group (n=10), which underwent the same hormonal stimulation protocol as the OHSS group in addition to treatment with metformin (Boehringer, Buenos Aires, Argentina) (50 mg/kg body weight) for 20 consecutive from days 22 to 41. The OHSS animal model has been first described by Ujioka et al. [23] and later used by other authors including our group [4,24-26], showing that this model shows the main characteristics of the human syndrome.

All hormones were intraperitoneally administered, while metformin was administered orally. The following two series of experiments were performed 48 h after the hCG injections. In the first series, both ovaries from five rats in each group were weighed and then homogenized for immunoblot analysis and VEGF level measurement. The blood was used for hormone assays and VEGF level determination. In the second series of experiments, one ovary from five rats in each group was used to measure ovarian vascular permeability with Evans Blue (EB), and the other was used for immunohistochemistry and follicular counting.

### Evaluation of vascular permeability

Vascular permeability was measured by monitoring the release of intravenously injected Evans Blue dye (EB) into the peritoneal fluid and ovarian tissue as previously described [24,27]. Briefly, 48 hours after hCG administration, 5 mM EB dye was diluted in distilled water and 0.2 ml was intravenously (i.v.) injected with an insulin injector through the tail vein. After a 30-min waiting period, all rats were weighed and intramuscularly administered 40/4 mg/kg ketamine (Ketafine; Brouwer, Argentina) / xilacine (Xylacine; Alfasan Woerden-Holland, Holland). Then, the peritoneal cavity was filled with 5 ml 0.9% NaCl solution and massaged for 30 seconds, and subsequently, the fluid was gently extracted from the abdominal cavity to prevent tissue or vessel damage. Protein interference was avoided by recovering the peritoneal fluid in tubes containing 0.05 ml 0.1 N NaOH. Fluid was clarified by centrifugation at 900 × *g* for 12 min, and then it was used to determine the EB concentration in the recovered fluid. Then, euthanasia was performed, and the ovaries were removed. One ovary was removed and fixed in % (w/v) formaldehyde, and the other ovary was incubated in 2 ml of formamide for 24 hsh at 37°C.

The EB concentration in the formamide extract and in peritoneal fluid was measured as a function of light absorption at 600 nm, which was determined using a spectrophotometer. The EB concentration was expressed as micrograms per mL of the peritoneal fluid, and the ovarian EB content was expressed as nanograms per milligram tissue wet weight.

### Hormone assays

Serum estradiol levels were measured using an electrochemiluminescene immunoassay (ECLIA) according to the manufacturer’s instructions (Elecsys analyzer Roche Diagnostics), and serum progesterone levels were measured by an immunochemiluminescence assay (ICMA). All samples were measured at the same time to minimize errors. Results are expressed as nanograms per dL serum for P_4_, and for E_2_, they are expressed as picograms per serum milliliter.

### Immunohistochemistry

Fixed ovarian samples were dehydrated and embedded in paraffin. Six-micron sections were mounted on gelatin-coated glass slides, deparaffinized in xylene, hydrated through a series of graded alcohols and washed in phosphate-buffered saline (PBS). The tissue sections were treated with 3% hydrogen peroxide (H_2_O_2_) to quench endogenous peroxidase activity. Nonspecific binding sites were blocked by treating the tissues with 5% (w/v) non-fat milk at room temperature for 30 min and subsequently incubated with primary antibodies for 24 hours at 4°C in a dark, moist chamber. We used a rabbit anti-NOS (ab-15203, Abcam, Cambridge, Mass, USA) antibody. After incubation with primary antibody, the sections were washed with PBS and treated with the appropriate biotinylated antibody (Vector Laboratories, Burlingame, United Kingdom) followed by an avidin-horseradish peroxidase-biotin complex (Vectastatin Elite ABC kit; Vector Lab, Burlingame, Calif, USA). The color reaction was visualized by exposure with the 30, 30-diaminobenzidine tetrahydrochloride DAB staining kit (Dako Cytomation, Carpinteria, CA, USA). Negative control sections were performed by omitting primary antibody. After color development, sections were counterstained with Mayer’s hematoxylin solution (Dako). Images from a standard light microscope were acquired with the Cool-SNAP-Pro Color camera system and analyzed with Image ProPlus 6.2 software (Media Cybernetics, Bethesda, MD).

### Ovarian histology and follicle counting

Ovarian tissue sections were stained with Masson trichrome stain and used to show the smooth muscle according to standard protocols and analyzed under a Zeiss Axiophot light microscope. Six-micron sections were mounted at 50 μm intervals onto microscope slides to prevent counting the same structure twice, according to the method described by Woodruff et al. [28]. Follicles were classified into three types: Preantral (PFs), antral (AFs) and preovulatory (POs). In the PFs group we gathered all the follicles that had no antrum, including primordial, primaries and preantral follicles strictly speaking. Primordial follicles were characterized as oocytes surrounded by a single layer of flattened granulosa cells. Primary follicles were characterized as oocytes surrounded by a single layer of cuboidal granulosa cells. Preantral follicles were characterized as oocytes surrounded by two or more layers of cuboidal granulosa cells with no visible antrum.

AFs were classified according to the presence of a small antrum and POs according to the presence of a big central antrum showing an eccentric oocyte. In addition, the number of corpora lutea (CL) was counted in each section analyzed. To assess the ovulation rate in the ovarian tissue, the number of corpora lutea was determined in the same sections as the other types of follicles. The abundance of each type of follicle was normalized by the total ovarian area in the section as reported previously [29,30]. The ovary area was measured with Image J (version 1.42q) and expressed per 10 mm^2^.

### Ovarian homogenates

Ten ovaries from each group were lysed for 20 min at 4°C in lysis buffer (20 mM Tris–HCl, pH = 8.0, 137 mM NaCl, 1% Nonidet P-40 and 10% glycerol) supplemented with 1 X protease inhibitors cocktail (Sigma). The lysate was centrifuged at 4°C for 10 min at 10 000 × *g*, and the pellet was discarded. Protein concentrations in the supernatant were measured by Bradford assay (Bio-Rad, Hercules, CA, USA).

### Determination of serum on ovarian VEGF levels

VEGF-A concentration was measured by commercially available Sandwich Enzyme-Linked Immunosorbent assay kit for rat VEGF (R & D System, Minneapolis, USA) using serum and ovarian homogenates. Assay was performed according to the manufacturer’s instruction. The detection sensitivity range of the kit was 30–2000 pgml^−1^ and showed no cross-reactivity with a series of soluble immunoactive molecules with a good reproducibility. The coefficient of variation was less than 9.5%. Results are expressed as pg/mg of tissue wet weight or pg/mL peritoneal fluid.

### Western blot

After boiling for 5 min the ovarian lysates, 90 μg of protein from each sample was applied to an SDS-polyacrylamide gel (10%) and electrophoresis was performed at 100 V for 1.5 h. The separated proteins were transferred onto PDVF membranes in transfer buffer (20% methanol, vol/vol; 0.19 M glycine; 0.025 M Tris-Base, pH = 8.3) for 1 h at 4°C. Blots were blocked for 1.5 h in TBS (4 mM Tris–HCl, pH = 7.5, 100 mM NaCl) containing bovine serum albumin (0.1%) at room temperature. Rabbit polyclonal anti-COX2 (1:500, overnight). Santa Cruz Biotechnology Inc., USA) and Glyceraldehyde-3-PDH (GADPH) (Millipore, USA) were used as primary antibodies. Rainbow-colored protein mass markers (14.3–200 kDa, Bio-Rad) were applied to determine the bands of COX2 (72 kDa) and GADPH (38 kDa). Protein bands were visualized by incubating the blots with biotin-conjugated secondary anti-rabbit IgG (1:2000, 1 h) followed by streptavidin–peroxidase complex and diaminobenzidine solution. Consistency of protein loading was evaluated by staining the membranes with Ponceau-S and relative to the GADPH protein levels. The intensities (area × density) of the individual bands on western blots were quantified by densitometry (Model GS-700, Imaging Densitometer, Bio-Rad). The experiment was independently repeated three times. Results are expressed in arbitrary units.

### Statistical analysis

Results are expressed as mean ± SEM. Comparisons between groups were performed using either one-way ANOVA in conjunction with Tukey’s test or Student’s *t*-test, where appropriate. The statistical level of significance was defined as *P* < 0.05.

## Results

### Measurement of body, ovarian and uterine weight and serum hormone levels

The effects of hyperstimulation and metformin on the body, ovarian and uterine weight and serum estradiol and progesterone are summarized in Table 1. Body weights were increased in hyperstimulated animals (OHSS group) with respect to control rats (p<0.001), and pretreatment with metformin (OHSS+MET) prevented this effect. The ovarian and uterine weights of the OHSS rats were greater than the control rats (p<0.001 and 0.05, respectively), while treatment with metformin did not modify these parameters.

**Table 1:** Body weight and endocrine characteristics of control and hyperstimulated animals

	**Control**	**OHSS**	**OHSS + M**
Body weight (g)	149,2 ± 1,0	167,8 ± 3,1***	156,2 ± 3,9#
Ovarian weight (mg)	63,2 ± 2,4	224,0 ± 13,1***	233,3 ± 15,4***
Uterine weight (mg)	231,6 ± 15,3	322,6 ± 21,9*	362,5 ± 29,9**
Progesterone (ng/dL)	188,7 ± 26,3	1035 ± 122,9***	751,1 ± 115,7**
Estradiol (pg/mL)	36,3 ± 0,7	166,3 ± 23,0***	98,5 ± 22,3#
n	10	10	10

Values are expressed as mean ± S.E.M and the *P* values were determined by one way ANOVA, followed by Tukey’s test. *P<0,05, **P<0,01 and ***P<0,001 respect to control group; ^#^ P<0,05 respect to the OSSH group.

The progesterone and estradiol levels were greater in OHSS rats compared with control animals (p<0.001), and metformin prevented the increase in estradiol levels.

### Ovarian morphology

**Figure 1:**
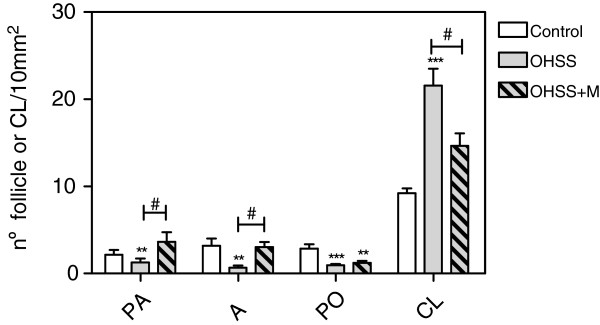
**Effect of the treatment with Metformin on the ovarian morphology in an OHSS rat model.** This is a quantitative morphometric analysis of the ovarian sections after Masson trichrome staining. Follicles were classified according to the stage of development indicated in materials and methods. Values are expressed as the number of follicles per 10 mm^2^. Data represent the mean ± S.E.M for 10 ovaries from different animals with the same treatment, and each value represents the mean of 10 sections from the same ovary. ** P< 0.01 and *** P< 0.001 respect to the control group; ^#^ P< 0.05 between the pointed groups.

To study the effects caused by metformin on ovarian morphology in the OHSS rat model, ovarian tissue sections were stained to determine the number of follicles in follicle stages (Figure 1). In the OHSS group, the number of preantral, antral and preovulatory follicles were significantly lower than that in the control group, while the number of CLs were higher in the OHSS group. Metformin significantly increased the number of preantral and antral follicles in treated compared with the untreated OHSS group. Moreover, treatment with metformin normalized the number of CLs.

### Effects of OHSS and metformin on vascular permeability

**Figure 2:**
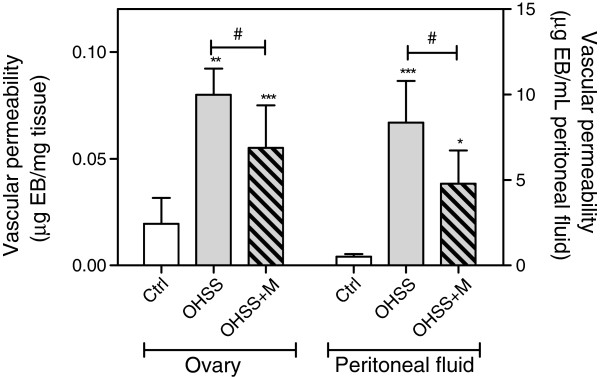
**Vascular permeability measured as concentration of Evans Blue (EB) in ovaries homogenates and in peritoneal fluid.** Each bar represents the mean ± S.E.M and the *P* values were determined by one way ANOVA, followed by Tukey’s test. * P< 0.05, ** P< 0.01 and *** P< 0.001 respect to the control group; ^#^ P< 0.05 between the pointed groups.

We were interested in evaluating the effects of treatment on vascular permeability because this is the main characteristic of the syndrome, and it is unknown whether metformin is able to prevent the increase in the capillary permeability. In our results, the ovarian EB content was significantly higher in hyperstimulated rats than control animals (p<0.01), indicating a significant increase in vascular permeability in this group; this effect was partially prevented by pre-treatment with metformin (OHSS vs. OHSS+MET, p<0.05) (Figure 2). The same effect was observed when we analyzed the peritoneal fluid EB content, where we found an increase in peritoneal fluid EB content in hyperstimulated rats (p< 0.001) that was partially prevented by pretreatment with metformin (OHSS vs. OHSS+MET, p<0.05).

### Effect of OHSS and metformin on VEGF levels

Because VEGF is the main factor involved in the pathology of OHSS, the level of this glycoprotein was measured in peripheral serum and the ovaries.

The ovarian VEGF concentration was significantly increased in the OHSS group with respect to the control group (p<0.001), and metformin partially reduced the VEGF concentration in treated compared with untreated OHSS rats (OHSS vs. OHSS+MET, p<0.05).

**Figure 3:**
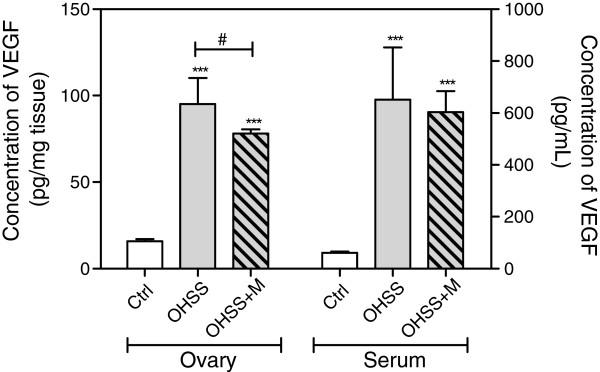
**Concentration of the vascular endotelial growth factor (VEGF) in ovaries homogenates and in serum.** Each bar represents the mean ± S.E.M and the *P* values were determined by one way ANOVA, followed by Tukey’s test. *** P< 0.001 respect to the control group and # P< 0.05 between the pointed groups.

The serum VEGF concentration measured in OHSS rats showed an important increased in comparison with controls (p<0.001), but metformin administration did not show significant changes in this parameter (Figure 3).

### Ovarian cyclooxygenase protein expression

**Figure 4:**
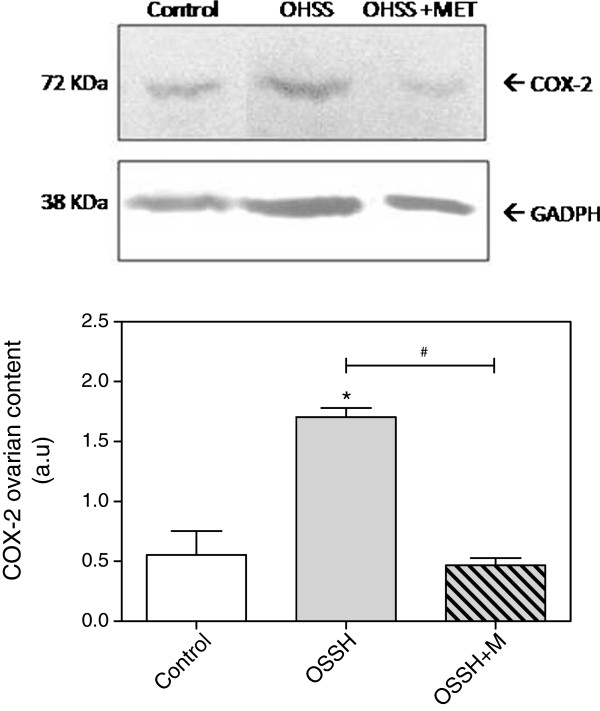
**Protein expression of Cycooxygenase type 2 (COX-2) in the ovaries.** Each bar represents the mean ± S.E.M and the *P* values were determined by one way ANOVA, followed by Tukey’s test. * P< 0.05 respect to the control group and ^#^ P< 0.05 between the pointed groups.

COX-2 is another important angiogenic factor that acts through VEGF that is one of the most important proteins involved in ovulation. Thus, because we are inducing ovulation and found that the VEGF levels are altered after treatment, we decided to evaluate the ovarian COX-2 protein expression. We found an increase in COX-2 ovarian levels in OHSS rats (p<0.05) that was prevented when the animals received pretreatment with metformin (OHSS vs. OHSS+MET, p<0.05) (Figure 4).

### NOS protein localization

The results of immunohistochemical analysis revealed moderate NOS staining in theca,granulose cells and oocytes from developed follicles in all groups analyzed.

**Figure 5:**
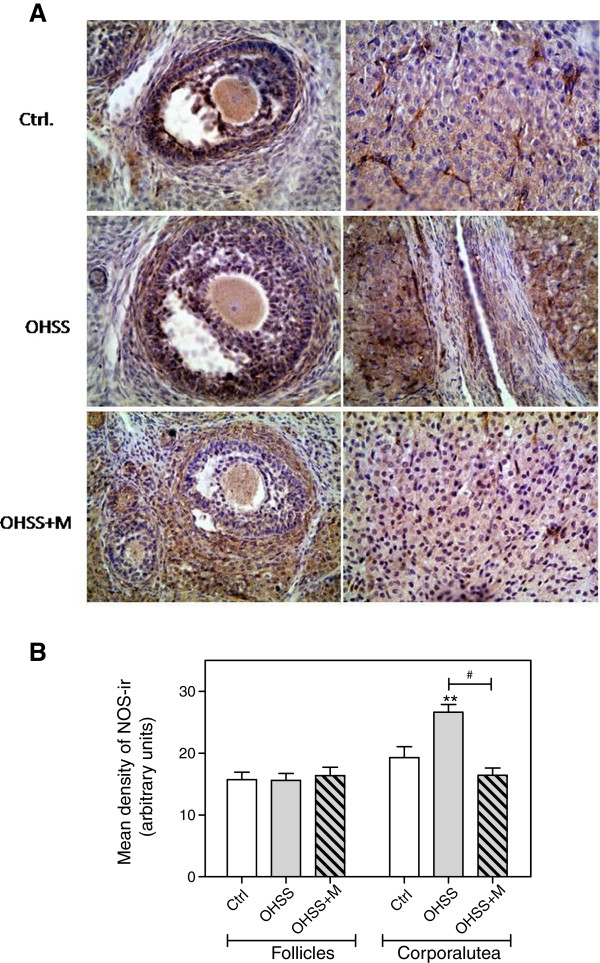
**NOS immunoreactivity in control and treated rat ovaries. (A)** Representative ovarian sections showing the immunolocalization of NOS from: upper panel: a control rat; middle panel: a OSSH rat; and lower panel: a OHSS+MET rat. A, C and E shows developed follicles (×100) while B, D and F (×100) shows corpora lutea. **(B)** Densitometric analyses of NOS immunoreactivity in the rat ovaries. Each bar represents the mean ± S.E.M and the Pvalues were determined by one way ANOVA, followed by Tukey’s test. ** P< 0.01 respect to the control group and #P< 0.0001 between the pointed groups.

Ovaries from the OHSS group showed intense NOS immunoreactivity in the corpus luteum (CL) when compared with CLs from the control group (p<0.01). In the OHSS+MET treatment group, NOS immunoreactivity was diminished in CLs when compared with those in the OHSS group (p<0.0001) (Figure 5), suggesting that metformin affects NOS protein expression.

## Discussion

Preventing OHSS remains a challenge for *in vitro* fertilization. Several strategies have been proposed for preventing the development of OHSS [31-38] as well as some drugs, such as oral contraceptives, leuprolide acetate, metformin, dexamethasone, low-dose aspirin and dopamine agonists. These drugs have been proven to be safe for other uses and are highly effective adjuncts for ovarian stimulation in “orphan” indications [39]. In particular, metformin administration has been confirmed to be a safe and effective strategy for reducing the incidence of OHSS [37]. Recently, it has been clinically demonstrated that it has been specified from a clinical point of view that metformin administration decreases the risk of moderate/severe OHSS by 80% and the risk of hospitalization by 75% [22]. Furthermore, patients with PCOS who are subfertile receive gonadotrophins while being treated with metformin to reduce the risk of OHSS [40]. However, at present,metformin specific effects and mechanisms of action are still unknown.

In the results of this study, we found an increase in the number of CLs in the OHSS group showing an increase in the induced ovulation rate (Figure 1). This effect was found concomitantly with a reduction in the number of preantral, antral and preovulatory follicles, suggesting that hormonal administration induced ovulation without inducing early follicular development. In addition, treatment with metformin prevented the effects of hormonal administration because the number of CLs was normalized in the OHSS+M group, indicating that metformin acts by decreasing the ovulation rate. The fact that metformin normalized the ovulation rate and number of preantral and antral follicles suggests that metformin was capable of affecting early folliculogenesis and ovulation. This result is in concordance with the that described by De Leo [41] who found a reduction in the number of follicles>15 mm in diameter in patients who received metformin treatment before the induction of ovulation. This finding indicates a lower incidence of ovarian overstimulation after metformin administration and supports the hypothesis that insulin plays a role in the endocrine and paracrine control of the ovaries [41].

Research suggests that VEGF is responsible for the increased vascular permeability leading to the extravasation of protein-rich fluid and subsequent OHSS progression [42,43]. In addition, high levels of VEGF have been detected in serum and follicular fluids from women with severe OHSS [44]. Here, we found increased VEGF levels in the ovaries and serum from rats with induced OHSS. Moreover, in this OHSS group, we detected one of the main characteristics of the syndrome: increased vascular permeability, which was demonstrated by the increased level of Evans Blue dye detected in different organs. In our results, this increased permeability was reduced with the metformin treatment (Figure 2). The increase in vascular permeability in the OHSS animals may be caused by the high levels of VEGF observed in this group as previously described [45,46]; however, further experiments are needed to clarify this point. Furthermore, it has been described in another system that metformin prevented the increased vascular permeability [47]. The fact that metformin prevented the increase in vascular permeability and the levels of VEGF (Figure 3) suggests that the mechanism by which metformin normalizes the vascular permeability may be through VEGF modulation.

In addition to VEGF, cyclooxygenase 2 (COX-2) has been described as an angiogenic factor in other systems [48,49]. COX-2 is a key enzyme in the conversion of arachidonic acid to prostaglandins and other eicosanoids. COX-2 is induced by a variety of factors, including cytokines, growth factors, and tumor promoters, and it is upregulated in many cancers and has been associated with increased VEGF production and angiogenesis [50]. Moreover, the vascular permeability in mouse skin induced by VEGF, was mediated by modulation of NOS and COX-2 [7]. Although the importance of COX-2 in the ovulatory process has been documented, the exact functions of this prostaglandin in human ovaries, fertilization, and implantation are not completely understood [43].

In our results, we found an increase in the ovarian COX-2 expression in the OHSS group concomitantly with an increase in ovarian NOS levels. Our findings agree with previous reports that showed an increase in ovarian COX-2 expression in a mild OHSS condition [4] and suggest that, in our model, VEGF may be acting through COX-2 and NOS in the ovary.

In contrast, we found that metformin restored vascular permeability and normalized the COX-2 and the VEGF ovarian levels. The effect of metformin in preventing the increase in ovarian COX-2 expression has also been observed in an animal model of PCOS [19]. Moreover, the effect of metformin in decreasing VEGF levels has been also described in other systems [51,52]. Taking into account these findings, we suggest that, in our model, metformin was able to normalize vascular permeability by normalizing the VEGF abundance and may be acting through COX-2 and NOS. An important regulator of these angiogenic molecules is hypoxia-inducible factor-1 (HIF-1). This transcriptional complex is expressed in rat ovarian granulosa cells in response to hCG treatment, and the inhibition of HIF-1 inhibits ovulation in rats [53]. HIF-1 increases the transcription of several genes to produce proteins that promote blood flow and inflammation including VEGF, NOS and COX-2 [54].

Further studies are being designed to clarify whether metformin normalizes vascular capillarity directly by affecting ovarian COX-2 and VEGF abundance or if metformin only decreases ovarian COX-2 levels, whether this protein normalizes vascular capillarity by regulating VEGF levels, and to elucidate whether HIF-1 is involved in this interplay.

In conclusion, our results demonstrate that metformin has a beneficial effect on OHSS by reducing increasing body weight, circulating progesterone and estradiol and vascular permeability. These effects are mediated by a metformin-mediated inhibition of the vasoactive molecules VEGF, COX-2 and partially NOS, which are molecules increased in OHSS and responsible for a variety of symptoms related to OHSS.

## Competing interests

The authors declared that they have no competing interest.

## Authors’ contributions

EME, RQ, DAP and CP were responsible for the conceptualization of the work, data interpretation and manuscript preparation. EME, RQ, CC VB and GRV assisted in data collection and all the experiments. DAP and CP designed and supervised the study. All authors read and approved the final manuscript.
